# Endoscopic spectrum of autoimmune gastritis across disease stages: a focus on Barrett’s esophagus and ME-NBI features

**DOI:** 10.3389/fimmu.2026.1765070

**Published:** 2026-06-03

**Authors:** Zhongmian Zhang, Jingna Tao, Liju Zhang, Lan Wang, Jiaqi Wang, Jinglin Zhang, Rui Han, Zhihong Li

**Affiliations:** 1 Digestive Disease Center, Beijing Hospital of Traditional Chinese Medicine, Capital Medical University, Beijing, China; 2 Department of Gastroenterology, Dongzhimen Hospital, Beijing University of Chinese Medicine, Beijing, China; 3 Department of Rehabilitation, Second Affiliated Hospital of Heilongjiang University of Chinese Medicine, Harbin, China

**Keywords:** autoimmune gastritis, Barrett esophagus, endoscopy appearance, magnifying endoscopy with narrow-band imaging, remnant oxyntic mucosa

## Abstract

**Background and aim:**

Autoimmune gastritis (AIG) is an immune-mediated disorder characterized by corpus-predominant atrophy. While serological markers are useful for screening, they inadequately reflect the dynamic progression of mucosal atrophy. Endoscopy remains the diagnostic cornerstone, yet a systematic analysis of its features across AIG stages is lacking. This study aimed to comprehensively analyze the clinical and endoscopic characteristics of AIG and evaluate the potential of endoscopic manifestations in defining disease stages, with particular focus on the intriguingly high prevalence of Barrett’s esophagus (BE).

**Methods:**

In this retrospective study, 52 Chinese patients fulfilling both serological (positivity for anti-parietal cell and/or anti-intrinsic factor antibodies) and endoscopic/histopathological criteria for AIG were enrolled. Patients were classified into early-stage (remnant oxyntic mucosa, ROM >10%) or late-stage (ROM ≤10%) AIG. White light endoscopy (WLE) and magnifying endoscopy with narrow-band imaging (ME-NBI) were used to evaluate a comprehensive set of endoscopic features.

**Results:**

The cohort had a mean age of 56.68 years and a female predominance (61.5%). Hypergastrinemia was present in 82.5% of patients, with significantly decreased pepsinogen I levels and pepsinogen I (PG I)/pepsinogen II (PG II) ratio (PGR). Late-stage AIG exhibited significantly lower levels of PG I and PG II compared to early-stage (P = 0.05 and P = 0.04, respectively). Endoscopically, sticky adherent dense mucus (51.9%) and swelling of areae gastricae (55.8%) were common WLE findings. ME-NBI revealed a high frequency of pseudopyloric metaplasia (75%). Notably, BE was detected in 73.08% of patients, with short-segment BE (SSBE) being more frequent in early-stage (60.9% vs. 31.0%, P<0.05) and long-segment BE (LSBE) in late-stage disease (37.9% vs. 17.4%). Features suggesting advanced atrophy, such as cast-off skin appearance (CSA) and white globe appearance (WGA), were more prevalent in late-stage AIG (P = 0.03 for both). However, in multivariate analysis, no single endoscopic feature emerged as an independent predictor of AIG stage.

**Conclusions:**

The endoscopic landscape of AIG is diverse and evolves with disease progression. The high prevalence of BE, particularly its association with disease stage, challenges the conventional acid-reflux pathogenesis and suggests a role for duodenogastric reflux in the context of achlorhydria. While no single endoscopic feature can definitively stage AIG, a multimodal assessment combining WLE and ME-NBI provides valuable insights for diagnosis and stratification.

## 1 Introduction

Autoimmune gastritis (AIG) is an immune-mediated disorder driven by autoantibodies targeting the parietal cell H^+^/K^+^-ATPase, resulting in parietal cell loss, achlorhydria, and compensatory hypergastrinemia (1–3). Morphologically, it is defined by corpus-predominant atrophy with relative antral sparing—a pattern distinct from Helicobacter pylori (HP)-associated gastritis, which typically initiates in the antrum and progresses proximally (4). This “reverse atrophy” is a hallmark endoscopic feature. Serologically, AIG is associated with anti-parietal cell antibodies (APCA) and anti-intrinsic factor antibodies (AIFA), which contribute to vitamin B_12_ malabsorption and may lead to pernicious anemia (5).

The clinical significance of AIG extends beyond anemia; it confers an elevated risk of gastric adenocarcinoma and neuroendocrine tumors due to sustained atrophy and hypergastrinemia. Moreover, its frequent association with other autoimmune conditions underscores its systemic nature (6–8).

Although serological markers such as APCA and gastrin have certain roles in screening for AIG, they cannot accurately reflect the degree of mucosal atrophy or its dynamic progression (9–11). Therefore, endoscopic examination remains the gold standard for diagnosis and staging. The recognized endoscopic features include atrophy mainly in the gastric body, thick mucus, pseudopolyps of residual gastric acid secretion mucosa, and loss of folds (12–17). Advanced imaging techniques, especially magnifying endoscopy with narrow-band imaging (ME-NBI), have further revealed subtle features such as castoff skin appearance (CSA), white globe appearance (WGA), and pseudo-pyloric metaplasia (16, 18, 19).

Although these findings provide new clues for AIG’s endoscopic diagnosis, the current research mainly focuses on describing individual or a few features, lacking a systematic and comprehensive analysis of the entire endoscopic range. In particular, during the progression of AIG from the early stage to the late stage, there are differences in its endoscopic manifestations and serological indicators. Moreover, in this study, when we sorted and analyzed the endoscopic manifestations of AIG patients, we discovered an interesting phenomenon: the occurrence frequency of Barrett’s esophagus (BE) is relatively high. This poses a challenge to the traditional acid reflux theory of BE pathogenesis. Is Barrett’s esophagus related to AIG in some way? Is it related to the stage of AIG?

To address these shortcomings, this study aims to conduct a systematic retrospective analysis of the endoscopic manifestations of patients with AIG who have been diagnosed through serological and pathological methods using white light endoscopy (WLE) and ME-NBI. Our specific objectives are: (1) To explore the differences in serological and endoscopic manifestations across different stages of AIG; (2) To assess the differences in the detection rate of BE in AIG across different stages. Through the exploration of these contents, our goal is to reliably guide the diagnosis and staging of AIG through multi-dimensional endoscopic assessment, thereby providing a basis for more personalized monitoring and management strategies.

## 2 Methods

### 2.1 Study population and inclusion criteria

From January 2022 to June 2025, a retrospective analysis was conducted on 82 Chinese patients who met the predefined diagnostic criteria for AIG. To ensure standardized evaluation, the Committee of AIG Research (CARP) established consensus-driven criteria and endoscopic diagnostic guidelines, thereby minimizing interobserver variability. Eligible participants were required to fulfill the following conditions:

#### 2.1.1 Diagnostic confirmation

Presence of endoscopic and/or histopathological features consistent with AIG (3).

#### 2.1.2 Serological evidence

APCA tenfold or more is positive (3).

#### 2.1.3 Inclusion threshold

Only patients satisfying both diagnostic and serological criteria (1 + 2) were enrolled in the study.

### 2.2 AIG stage criteria

The remnant oxyntic mucosa (ROM) is a characteristic endoscopic finding of AIG. In this study, AIG stages was classified based on the area of ROM observed under endoscopy, while referencing the AIG atrophic stage (AIG-AS) (20), we propose that ROM quantification serves as a two-dimensional (‘area-based’) indicator for evaluating the degree and progression of gastric atrophy. In the present study, AIG early stage was defined as ROM>10%, AIG end stage was defined as ROM ≤ 10% due to the poor inter-observer agreement observed for ROM, which could lead to significant misclassification of absent AIG stage. The ROM was estimated independently by each physician using retrieved endoscopic images following the consensus meeting.

### 2.3 Assessment of endoscopic appearance

WLE evaluation encompassed the assessment of BE, white patches, sticky adherent dense mucus, swelling of areae gastricae, oxyntic mucosa pseudopolyp, diffuse redness, and xanthomas. Meanwhile, ME-NBI was employed to examine the following features: CSA, WGA, light blue crest (LBC), white opaque substance (WOS), swelling of marginal crypt epithelium, disappeared crypt pit, pseudopyloric metaplasia, and white short stick structures.

#### 2.3.1 BE diagnosis

**Figure 1:**
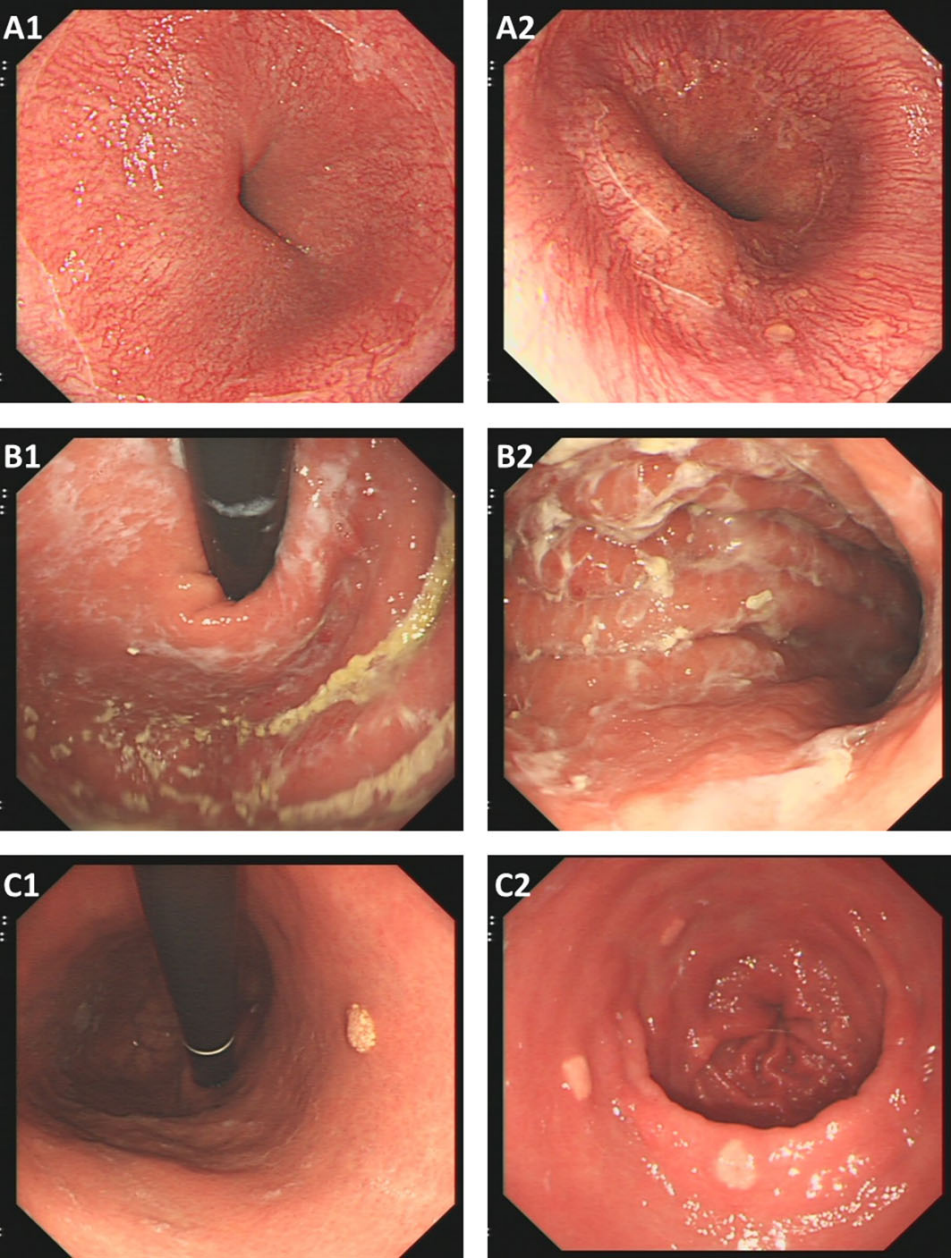
**| (A1)** LSBE (≥3 cm) and **(A2)** SSBE (1 and<3 cm). **(B1, B2)** Characteristic sticky adherent dense mucus with creamy white to yellowish appearance. **(C1, C2)** Typical pale yellow granular-like patchy protrusions.

As described earlier, after reaching diagnostic consensus for BE, participating physician retrospectively evaluated endoscopic images obtained at study enrollment to determine BE presence/absence and measure its length when present (21). The gastroesophageal junction was identified based on either the oral side end of gastric folds or the distal boundary of palisade vessels (22). Following Japanese Esophageal Society guidelines (23), BE was confirmed when columnar epithelium extended continuously from the junction into the esophageal lumen, regardless of segment length. Study participants were then categorized into four groups: absent, ultra-short-segment BE (<1 cm, USSBE), short-segment BE (1 and <3 cm, SSBE), and long-segment BE (≥3 cm, LSBE). Given the documented poor interobserver reliability in diagnosing USSBE and its potential for diagnostic inaccuracy (21), our analysis defined BE as columnar epithelium ≥1 cm, combining SSBE and LSBE (**Figures 1A1, A2**) cases. Consequently, to examine distinctive BE features without USSBE-related confounding, we excluded these marginal cases from risk factor analyses. Post-consensus meeting, each physician independently measured BE lengths using archived endoscopic images of the esophagogastric junction.

#### 2.3.2 Endoscopic appearance under WLE and ME-NBI

**Figure 2:**
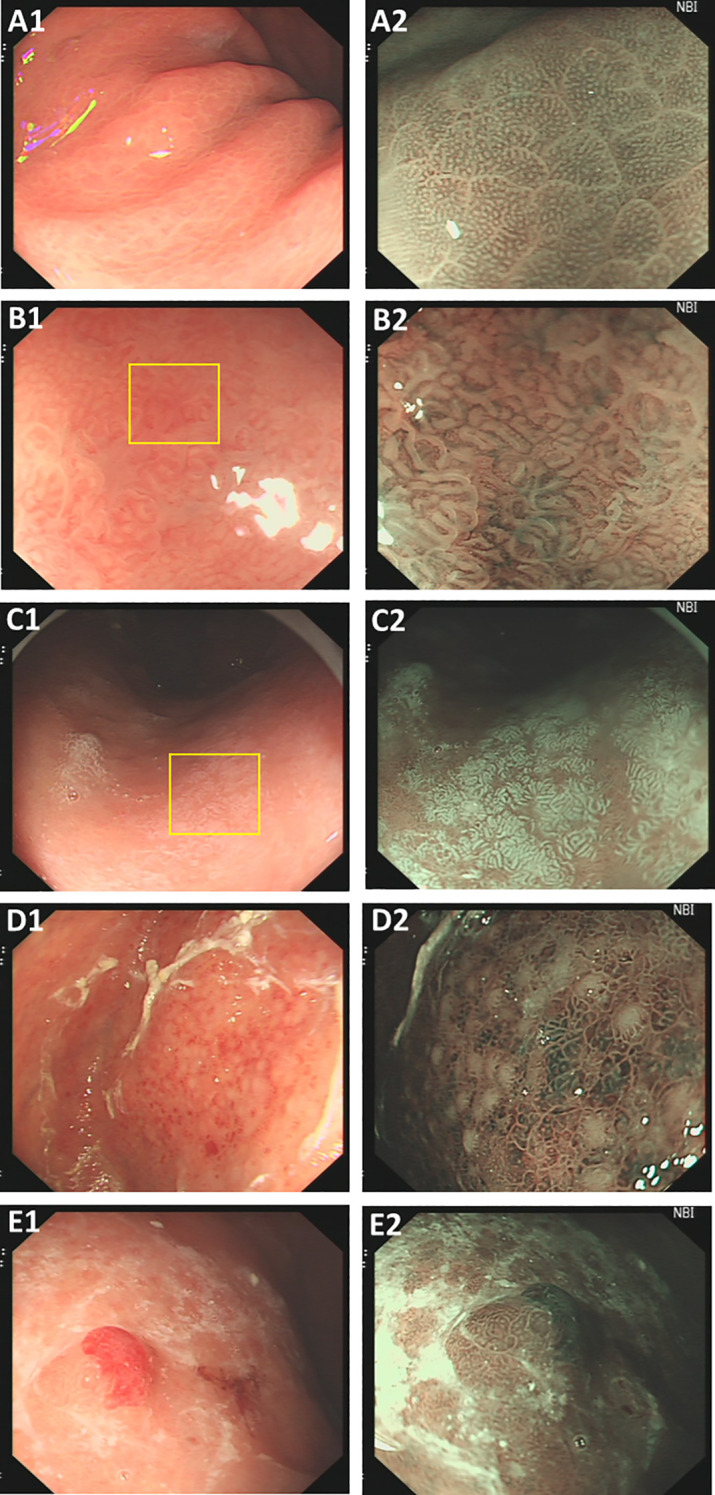
**| (A1)** fissure-like patterns. **(A2)** ME-NBI image of **(A1)** reveal a fish scale-like appearance. **(B1, B2)** pseudopyloric metaplasias. **(C1)** white patches. **(C2)** white rod-shaped intestinal metaplasia patches under ME-NBI. **(D1, D2)** diffuse redness. **(E1, E2)** oxyntic mucosa pseudopolyps.

Sticky adherent dense mucus exhibited a thick, creamy appearance with a yellowish-white hue and was tightly attached to the mucosa (**Figures 1B1, B2**). Swelling of areae gastricae results from inflammatory hyperplasia of the foveolar epithelium. Under WLE, it appears as fissure-like patterns, while NBI reveals a fish scale-like appearance (**Figure 2**).

**Figure 3:**
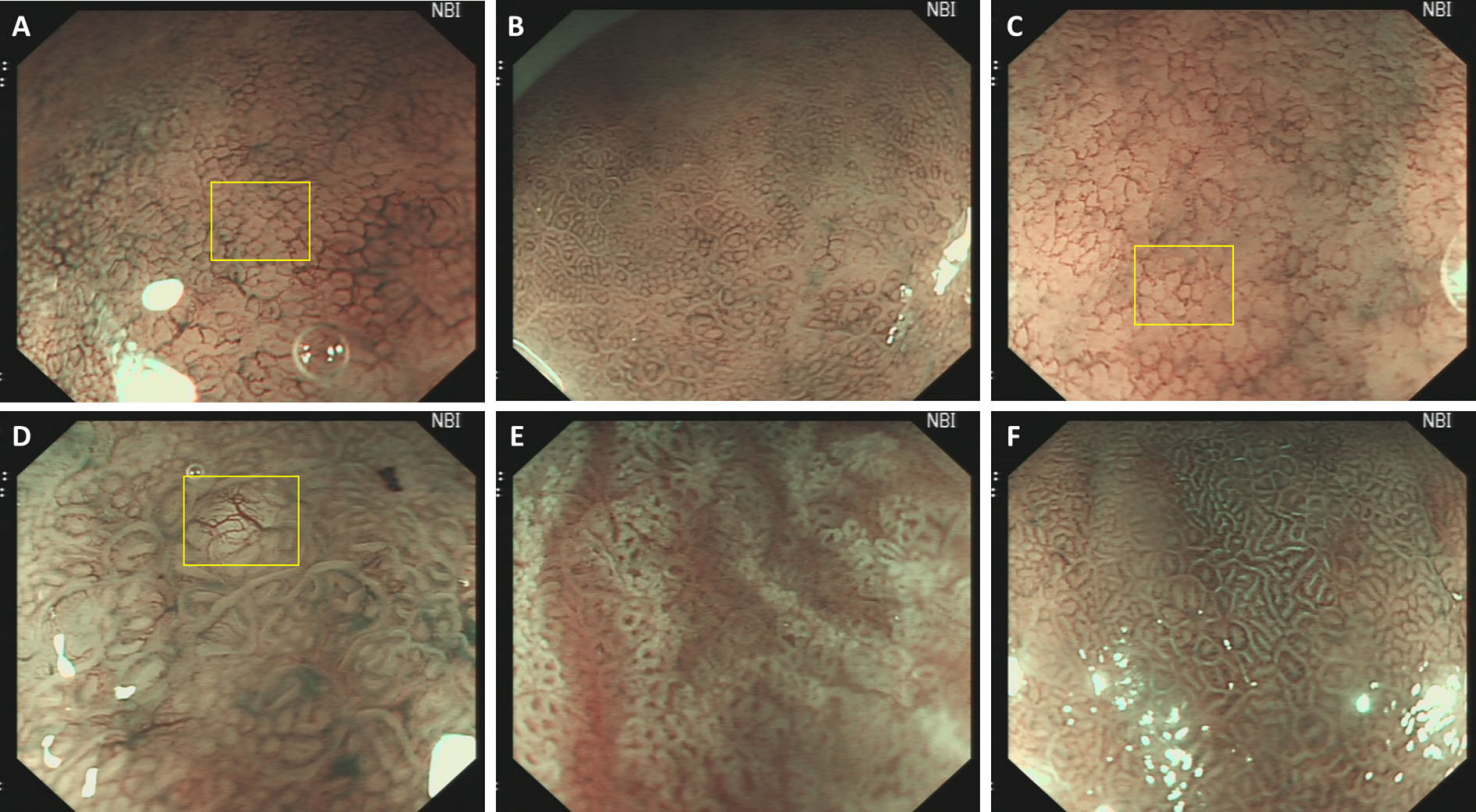
ME-NBI appearance. **(A)** marginal crypt epithelium swelling. **(B)** disappeared crypt pits. **(C)** CSA is characterized by the disappearance of the normal fundic pit surrounded by reticular capillaries. **(D)** the WGA pattern exhibited small, dome-shaped, pale mucosal elevations diffusely distributed throughout the gastric corpus, with overlying epithelial capillaries. **(E)** WOS. **(F)** LBC.

Pseudopyloric metaplasias (**Figure 2**), The WGA pattern exhibited small, dome-shaped, pale mucosal elevations diffusely distributed throughout the gastric corpus, with overlying epithelial capillaries. (**Figure 3D**), and CSA (24) is characterized by the disappearance of the normal fundic pit surrounded by reticular capillaries. (**Figure 3C**). Crypt pit architecture alterations included marginal crypt epithelium (MCE) swelling (**Figure 3A**) and disappeared crypt pits (**Figure 3B**). Rare ME-NBI features detected included WOS (**Figure 3E**), LBC (**Figure 3F**).

Additionally, we identified several rare endoscopic features, including white patches (**Figure 2C1**) under WLE and white rod-shaped intestinal metaplasia patches under ME-NBI (**Figure 2C2**), diffuse redness (**Figures 2D1, D2**), oxyntic mucosa pseudopolyps (**Figures 2E1, E2**), and xanthomas (**Figures 1C1, C2**).

### 2.4 Statistical analysis

The SPSS Statistics (Version 31.0, Armonk, NY: IBM Corp) was used for all computations. A 2-side P < 0.05 was considered statistically significant. More in detail, continuous data were described with mean ± standard deviation (mean ± SD), and categorical data as counts and percent, and compared using Fisher’s exact test or chi-square test as appropriate. Logistic regression models for AIG stages were fitted for estimating the Odds ratio (OR) associated with the candidate risk factors. A multivariable analysis was fully adjusted for PG I, PG II, BE, Swelling of areae gastricae, CSA, WGA.

## 3 Results

Initially, 82 patients were enrolled; however, 22 were excluded due to the lack of images from the magnifying endoscopy, and 5 were excluded due to extensive missing laboratory data. Ultimately, a total of 52 patients with AIG were included in the study.

### 3.1 Clinical characteristics of patients with autoimmune gastritis

**Table 1** shows the clinical characteristics features. The study included 52 patients with AIG, comprising 20 males (38.5%) and 32 females (61.5%), with a mean age of 56.68 ± 11.15 years. Among 32 patients tested for Helicobacter pylori Ab 9 (28.12%) were positive. Elevated gastrin levels were observed in 82.5% of patients (33/40), while pepsinogen I levels and PGR were significantly reduced. Anti-parietal cell antibodies were detected in 87.76% of cases (43/49). Vitamin B12 (586.50 ± 68.59 pg/ml) and folic acid (14.57 ± 2.79 ng/ml) levels remained within normal ranges.

**Table 1:** Clinical characteristics and endoscopic appearance of patients with autoimmune gastritis.

Clinical characteristics	
Age (mean ± SD)	56.68 ± 11.15
sex	
Male	20 (38.5%)
Female	32 (61.5%)
HP positive (n=32);	9 (28.12%)
G-17 (pmol/L (1.0-7.0); n=40)	33 (82.50%)
PG I (ug/L (>70.0); n=50)	34.60 ± 30.37
PG II (ug/L (<15.0); n=50)	14.01 ± 8.21
PGR	1.31 ± 0.42
APCA (n=49)	43 (87.76%)
Anti-thyroglobulin Ab positive (n=30)	10 (33.33%)
Folic acid (ng/ml (3.2-19.6); n=48)	14.57 ± 2.79
Vitamin B12 (pg/ml (180-916); n=50)	586.50 ± 68.59
Endoscopic appearance	
AIG-AS	
Early stage	23 (44.2%)
Late stage	29 (55.8%)
BE	
SSBE	23 (44.23%)
LSBE	15 (28.85%)
Indeterminable	14 (26.92%)
WLE	
White patches	4 (7.7%)
Sticky adherent dense mucus	27 (51.9%)
Swelling of areae gastricae	29 (55.8%)
oxyntic mucosa pseudopolyps	3 (5.8%)
Diffuse redness	4 (7.7%)
Xanthomas	1 (1.9%)
ME-NBI	
CSA	9 (17.3%)
WGA	15 (28.8%)
LBC	3 (5.8%)
WOS	4 (7.7%)
MCE swelling	14 (26.9%)
Disappeared crypt pits	7 (13.5%)
Pseudopyloric metaplasias	39 (75%)
White rod-shaped intestinal metaplasia patches	1 (1.9%)

AIG-AS: AIG-Atrophic stage; BE: Barrett's esophagus; WLE: White light endoscopy; ME-NBI:magnifying endoscopy with narrow-band imaging.

### 3.2 Endoscopic features

Endoscopic staging revealed 44.2% early-stage (n=23) and 55.8% late-stage AIG (n=29). Key findings included in **Table 1**.

WLE: Sticky adherent dense mucus (51.9%) (**Figures 1B1, B2**), swelling of areae gastricae (55.8%) (**Figure 2**), and SSBE (44.23%) (**Figure 1A2**) were most prevalent. Additionally, we identified several rare endoscopic features, including white patches (7.7%; **Figures 2C1, C2**), diffuse redness (7.7%; **Figures 2D1, D2**), oxyntic mucosa pseudopolyps (5.8%; **Figure 2E1, E2**), and xanthomas (1.9%; **Figures 1C1,C2**).

ME-NBI: Pseudopyloric metaplasias (75%; **Figures 2B1, B2**), WGA patterns (28.8%; **Figure 3D**), and CSA (17.3%; **Figure 3C**) were prominent. Crypt pit architecture alterations included MCE swelling (26.9%) (**Figure 3A**) and disappeared crypt pits (13.5%; **Figure 3B**). Rare ME-NBI features detected included WOS (7.7%; **Figure 3E**), LBC (5.8%; **Figure 3F**), and white short stick (1.9%; **Figure 2C1,C2**).

### 3.3 Comparative analysis by AIG stages

**Table 2** compare the laboratory, WLE, and ME-NBI depending on the AIG stages. Late-stage AIG showed significantly lower pepsinogen 1 (26.22 ± 23.28 vs 48.36 ± 50.88 μg/L, P = 0.05) and pepsinogen 2 levels (10.36 ± 3.88 vs 13.95 ± 7.65 μg/L, P = 0.04) compared to Early-stage.

**Table 2:** Comparison of the main laboratory, WLE, and ME-NBI depending on the AIG stages.

Continuous variables	Early stage	Late stage	*P* value
Age (mean ± SD)	61.43 ± 9.51	58.59 ± 10.42	0.31
G-17	18.69 ± 15.87	22.36 ± 23.16	0.57
PG I	48.36 ± 50.88	26.22 ± 23.28	0.05
PG II	13.95 ± 7.65	10.36 ± 3.88	0.04
PGR	3.54 ± 3.82	2.43 ± 2.29	0.21
Anti-thyroglobulin Ab	102.62 ± 152.29	346.48 ± 721.77	0.2
Folic acid (ng/ml)	15.93 ± 5.64	16.42 ± 5.58	0.76
Vitamin B12 (pg/ml)	552.75 ± 406.95	397.79 ± 248.04	0.1
Categorical variables			
**Sex**	stage early	late	0.29
Male	7	13	
Female	16	16	
**HP**			0.69
positive	4	5	
negative	12	11	
**APCA**			0.06
positive	18	25	
negative	5	1	
**BE**			<0.05
SSBE	14	9	
LSBE	4	11	
Indeterminable	5	9	
**WLE**			
White patches			0.42
positive	1	3	
negative	22	26	
Sticky adherent dense mucus			0.25
positive	14	13	
negative	9	16	
Swelling of areae gastricae			<0.05
positive	15	14	
negative	8	15	
Oxyntic mucosa pseudopolyps			0.42
positive	2	1	
negative	21	28	
Diffuse redness			0.81
positive	2	2	
negative	21	27	
Xanthomas			0.11
positive	2	0	
negative	21	29	
ME-NBI			
CSA			0.03
positive	1	8	
negative	22	21	
WGA			0.03
positive	3	12	
negative	20	17	
LBC			0.11
positive	0	3	
negative	23	26	
WOS			0.81
positive	2	2	
negative	21	27	
MCE swelling			0.61
positive	7	7	
negative	16	22	
Disappeared crypt pits			0.37
positive	2	5	
negative	21	24	
Pseudopyloric metaplasias			0.42
positive	16	23	
negative	7	6	
White rod-shaped intestinal metaplasia patches			0.37
positive	0	1	
negative	23	28	

HP: Helicobacter pylori; APCA: anti-parietal cell antibodies; BE: Barrett's esophagus; WLE: White light endoscopy; ME-NBI:magnifying endoscopy with narrow-band imaging.

### 3.4 Endoscopic appearance markers differed significantly

Barrett’s esophagus: SSBE predominated in early-stage (60.9% vs 31.0% in late-stage, P < 0.05), while LSBE was more frequent in late-stage (37.9% vs 17.4%). (**Figure 1**).

WLE: The presence of swelling of areae gastricae showed a statistically significant association with AIG progression stages (P < 0.05). Among patients exhibiting this feature (n=29), the distribution between early and late stages was nearly equivalent (early: 15 (51.7%) vs late: 14 (48.3%)). In contrast, patients without mucosal swelling (n=23) demonstrated a predominance of late-stage cases (15 (65.2%)) compared to early-stage presentations (8 (34.8%)). This inverse distribution pattern suggests potential diagnostic value of swelling of areae gastricae in staging assessment.

ME-NBI: CSA (8/29 vs 1/23, P = 0.03) and WGA (12/29 vs 3/23, P = 0.03) were more common in late-stage.

### 3.5 Multivariate logistic regression

**Table 3** summarizes the multivariable-adjusted odds ratios for AIG stages. Multivariable logistic regression analysis revealed no statistically significant associations between the investigated factors and the outcome of interest. PG I showed a neutral effect (OR = 1.00, 95% CI: 0.98-1.02, P = 0.81), while PG II demonstrated a non-significant negative association (OR = 0.91, 95% CI: 0.79-1.06, P = 0.22). Endoscopic findings of Barrett’s esophagus abnormalities (OR = 1.40, 95% CI: 0.63-3.13, P = 0.41), swelling of areae gastricae (OR = 0.61, 95% CI: 0.16-2.34, P = 0.47), CSA (OR = 6.68, 95% CI: 0.68-65.78, P = 0.10), and WGA (OR = 2.85, 95% CI: 0.57-14.23, P = 0.20) all failed to reach statistical significance, though CSA and WGA showed numerically elevated ORs.

**Table 3:** Logistic multivariable analysis looking at the OR of AIG stages after adjustment for potential confounders.

	OR	95% CI	*P* value
PG I	1.00	0.98-1.02	0.81
PG II	0.91	0.79-1.06	0.22
BE	1.40	0.63-3.13	0.41
Swelling of areae gastricae	0.61	0.16-2.34	0.47
CSA	6.68	0.68-65.78	0.10
WGA	2.85	0.57-14.23	0.20

## 4 Discussion

This study systematically analyzed the clinical characteristics and endoscopic manifestations of 52 patients with autoimmune gastritis. We analyzed the typical endoscopic manifestations and clinical features of autoimmune gastritis at different stages. Our aim was to explore the value of endoscopic features in the diagnosis and staging of autoimmune gastritis. More importantly, we discovered several WLE and ME-NBI features with potential diagnostic value.

### 4.1 Pathophysiological basis of AIG and correlation with clinical and endoscopic features

The cohort consisted of 52 AIG patients with a male-to-female ratio of 1:1.6, predominantly middle-aged or elderly, consistent with previous reports indicating a higher prevalence in females and individuals over 60 years, typically with a ratio of 1:2–3 (25). APCAs were positive in 87.76% of patients. Furthermore, 82.50% exhibited hypergastrinemia alongside significantly reduced PG I levels and PGR, reflecting the core pathophysiological hallmarks of AIG.

APCA serves as an early immunological marker, potentially appearing before symptom onset, with a general population prevalence of 2–5% (26). Our detection rate aligns with Lahner et al., who reported approximately 80% sensitivity and 90.3% specificity of APCA for AIG (10). The gradual decline in APCA titers during disease progression may be attributed to reduced antigen load as parietal cells are destroyed (11).

Pepsinogen acts as a “serological biopsy” for gastric mucosal function. PG I is primarily secreted by chief cells in the oxyntic glands, whereas PG II is produced by glands throughout the stomach (27). The pathogenesis of AIG involves an immune-mediated attack targeting parietal cells in the corpus and fundus, with the H+/K+-ATPase proton pump as the key autoantigen. This persistent process also leads to progressive chief cell loss, likely mediated by sensitized lymphocytes (28).

Consequent to the autoimmune destruction of parietal and chief cells, serum PG I levels and the PGR decline progressively. The loss of parietal cells results in achlorhydria and elevated gastric pH, which removes inhibitory feedback on antral G cells, leading to G cell hyperplasia and sustained hypergastrinemia (29). Hypergastrinemia may promote the development of gastric neuroendocrine tumors via stimulation of enterochromaffin-like cells. Serum gastrin is a crucial marker for AIG diagnosis and monitoring, with a proposed cutoff of 355 pg/mL demonstrating diagnostic utility (30). The combination of low PG I (or PGR) and elevated gastrin-17 has shown a sensitivity of 70.4% and specificity of 98.4% for diagnosing corpus atrophic gastritis (9).

HP antibodies were detected in 28.12% of tested patients. The role of HP in AIG pathogenesis remains contentious, with evidence suggesting dual potential roles as either a trigger or an inhibitor. Molecular mimicry between HP antigens and H+/K+-ATPase may initiate autoimmunity in genetically susceptible individuals (31), while some studies indicate that HP infection might conversely inhibit AIG progression via induction of Th2 responses (32). A recent meta-analysis suggested a negative correlation between HP infection and atrophic gastritis risk, possibly mediated by antral inflammation (33). Thus, the interplay likely depends on host factors and disease stage, warranting further investigation.

These pathophysiological alterations manifest endoscopically. Our observations diverge somewhat from the conventional consensus, revealing a diverse endoscopic spectrum in AIG. Sticky adherent dense mucus was observed in 51.9% of patients, likely resulting from severe mucosal atrophy and reduced gastric secretion, facilitating bacterial overgrowth under achlorhydric conditions (13). Oxyntic mucosa pseudopolyps, observed in 5.8% of cases, represent ROM islands amidst atrophy (34). Their morphology varies, with pseudopolyp-like lesions being a recognized pattern (19, 28). Kotera et al. described longitudinally arranged pseudopolyps with a “bamboo-joint” appearance as characteristic of early AIG (35).

Rare findings included white patches, diffuse redness, and xanthomas. Early AIG may present with mucosal edema, swelling of areae gastricae, and erythema, sometimes resembling a “salmon roe-like” appearance (35, 36). The white patches on WLE and corresponding White rod-shaped intestinal metaplasia patches structures on ME-NBI are particularly notable, representing, to our knowledge, the first report of such features in AIG.

On ME-NBI, pseudopyloric metaplasia was the most frequent finding (75%). This metaplastic change involves the transformation of fundic gland mucosa to an antral-type pattern, characterized microvasculature and crypt architecture alterations (37–39). Histologically, it reflects replacement by pseudopyloric or pyloric glands (3), and is reportedly more common than intestinal metaplasia in AIG (40, 41). Other ME-NBI features included MCE swelling (26.9%) and disappeared crypt openings (13.5%), indicative of glandular deformation and atrophy (42, 43).

Rare ME-NBI features such as WOS (7.7%) and LBC (5.8%), typically markers of intestinal metaplasia (44–46), were also observed. WOS presence correlates with higher gastric pH (47), consistent with the hypochlorhydric state in AIG. LBC is highly predictive of histological intestinal metaplasia (48, 49), and its appearance in AIG may correspond to late-stage disease accompanied by intestinal metaplasia.

### 4.2 High prevalence of Barrett’s esophagus in AIG: a novel association

A striking finding was the high prevalence of BE, endoscopically identified in 73.08% of patients (SSBE: 44.23%; LSBE: 28.85%). This prevalence markedly exceeds that in general non-Gastroesophageal reflux disease (GERD) (2.2%) or GERD (7%) populations (50), challenging the conventional acid-reflux paradigm for BE pathogenesis.

Traditionally, BE is regarded as a consequence of chronic acid exposure. However, AIG is characterized by hypochlorhydria, which would theoretically confer protection against acid-induced BE. This apparent paradox points to an alternative, non-acid-mediated pathogenic mechanism. We propose that duodenogastric reflux—rich in bile acids and other noxious agents—plays a primary role in the development of BE in patients with AIG (51–53). Achlorhydria compromises the stomach’s natural barrier against duodenal contents, thereby facilitating the reflux of weakly acidic or alkaline substances into the esophagus, where they can induce mucosal injury and subsequent metaplasia (54, 55).

Furthermore, the distribution of BE subtypes was correlated with the stage of AIG: SSBE was predominant in early-stage AIG (60.9% vs. 31.0% in late-stage, P < 0.05), whereas LSBE was more frequently observed in late-stage disease (37.9% vs. 17.4%). This progression from SSBE to LSBE may reflect prolonged exposure to noxious refluxate as gastric atrophy advances. Concomitant hypergastrinemia in late-stage AIG (56), known to act as a trophic factor (57), may further promote proximal extension of the metaplastic epithelium, thereby facilitating the development of LSBE (58). Experimental studies have confirmed that weakly acidic solutions can induce esophageal mucosal injury comparable to that caused by acidic reflux (59). These findings highlight the necessity of thorough esophageal evaluation in AIG patients, particularly those with advanced atrophy, to screen for BE and associated dysplastic changes.

### 4.3 Dynamic evolution of serological and endoscopic features with AIG progression

Late-stage AIG patients exhibited significantly lower levels of both PG I (p=0.05) and PG II (p=0.04) compared to early-stage patients. While early AIG primarily affects the corpus (reflected by decreased PG I), advanced disease with extensive atrophy beyond the corpus leads to a significant decline in PG II. Thus, a falling PGR signals corpus atrophy onset, whereas a subsequent decrease in PG II may indicate generalized and severe atrophy, marking progression to terminal stages.

Endoscopic findings also evolved with disease stage. Swelling of areae gastricae, the most common WLE finding (55.8%), demonstrated a unique association with staging. Its presence was similarly frequent in early and late AIG, but its absence was significantly associated with late-stage disease (65.2% of patients without swelling had late-stage AIG). This may reflect initial inflammatory edema in early AIG (60, 61), which subsides as atrophy predominates in later stages. Therefore, disappearance of mucosal swelling against an atrophic background may indirectly indicate progression.

ME-NBI features like CSA (more common in late-stage: 27.6% vs. early-stage, P = 0.03) and WGA (more common in late-stage, P = 0.03) signify severe glandular atrophy and destruction. CSA, characterized by preserved capillaries without visible glandular openings, suggests profound atrophy (16, 24). WGA, spherical white subepithelial lesions, is a specific marker for early gastric cancer but is also observed in AIG, potentially indicating increased neoplastic risk (18, 62). Their higher prevalence in late-stage AIG aligns with the natural history of progressive glandular loss.

### 4.4 Limitations of multivariate analysis: sample size and biological complexity

Although univariate analyses identified several stage-associated indicators, multivariate logistic regression failed to establish independent predictors of AIG stage. Notably, CSA (OR = 6.68) and WGA (OR = 2.85) exhibited high, albeit non-significant, odds ratios with wide confidence intervals, likely due to the limited sample size (n=52) reducing statistical power after adjustment for covariates. These features may still hold clinical relevance as markers of severity, but larger prospective studies are needed to confirm their independent predictive value.

This result addresses the question posed in our title: currently, no single endoscopic feature can reliably define AIG stages. Endoscopic diagnosis and staging require integrating clinical context, serological data, and a combination of WLE and ME-NBI findings to form a comprehensive diagnostic picture.

### 4.5 Study strengths, limitations, and future directions

A key strength of this study is the systematic integration of serology, WLE, and ME-NBI for a holistic assessment of AIG patients. We compared endoscopic features across disease stages, providing insights into the dynamic endoscopic evolution of AIG. A major novel contribution is the inclusion of Barrett’s esophagus as a significant finding in AIG, suggesting routine esophageal evaluation in these patients.

Limitations include the lack of detailed one-to-one histopathological correlation for each endoscopic finding. Future studies should incorporate systematic biopsies to validate these endoscopic features against the pathological gold standard. Our use of a binary staging system (early: ROM>10%; late: ROM ≤ 10%), while practical, simplifies the continuous disease spectrum. The Japanese AIG-AS three-stage system, though more detailed, suffers from inter-observer variability. Developing objective methods for ROM quantification remains a challenge.

As a retrospective single-center study, potential selection bias and limited generalizability exist. The modest sample size likely underpowered the multivariate analysis. Larger, multicenter prospective cohorts are necessary to validate our findings and explore the predictive value of specific endoscopic features further.

## Data Availability

The original contributions presented in the study are included in the article/Supplementary Material. Further inquiries can be directed to the corresponding author.
